# Interactive Sections of an Internet-Based Intervention Increase Empowerment of Chronic Back Pain Patients: Randomized Controlled Trial

**DOI:** 10.2196/jmir.3474

**Published:** 2014-08-13

**Authors:** Silvia Riva, Anne-Linda Camerini, Ahmed Allam, Peter J Schulz

**Affiliations:** ^1^Institute of Communication and HealthUniversità della Svizzera italianaLuganoSwitzerland; ^2^Università degli Studi di MilanoDepartment of Health SciencesMilanItaly

**Keywords:** Internet-based intervention, interactivity, patient empowerment, chronic back pain, health outcomes, decision, health, physical activity, pain burden, medication misuse, gamification

## Abstract

**Background:**

Chronic back pain (CBP) represents a significant public health problem. As one of the most common causes of disability and sick leave, there is a need to develop cost-effective ways, such as Internet-based interventions, to help empower patients to manage their disease. Research has provided evidence for the effectiveness of Internet-based interventions in many fields, but it has paid little attention to the reasons why they are effective.

**Objective:**

This study aims to assess the impact of interactive sections of an Internet-based self-management intervention on patient empowerment, their management of the disease, and, ultimately, health outcomes.

**Methods:**

A total of 51 patients were recruited through their health care providers and randomly assigned to either an experimental group with full access to the Internet-based intervention or a control group that was denied access to the interactive sections and knew nothing thereof. The intervention took 8 weeks. A baseline, a mid-term after 4 weeks, and a final assessment after 8 weeks measured patient empowerment, physical exercise, medication misuse, and pain burden.

**Results:**

All patients completed the study. Overall, the intervention had a moderate effect (*F*_1.52_=2.83*, P*=.03, η^2^=0.30, *d*=0.55). Compared to the control group, the availability of interactive sections significantly increased patient empowerment (midterm assessment: mean difference=+1.2, *P*=.03, *d*=0.63; final assessment: mean difference=+0.8, *P*=.09, *d*=0.44) and reduced medication misuse (midterm assessment: mean difference=−1.5, *P*=.04, *d*=0.28; final assessment: mean difference=−1.6, *P=*.03, *d*=−0.55) in the intervention group. Both the frequency of physical exercise and pain burden decreased, but to equal measures in both groups.

**Conclusions:**

Results suggest that interactive sections as part of Internet-based interventions can positively alter patients’ feelings of empowerment and help prevent medication misuse. Detrimental effects were not observed.

**Trial Registration:**

ClinicalTrials.gov: NCT02114788; http://www.clinicaltrials.gov/ct2/show/NCT02114788 (Archived by WebCite at http://www.webcitation.org/6ROXYVoPR).

## Introduction

### Chronic Back Pain

Chronic back pain (CBP) is one of the most highly prevalent medical conditions and represents a significant public health problem. It is the second most common pain after headache and it has been identified recently as the single most important among the principal causal factors of years lived with disability worldwide [1]. As recently evaluated by Dunn et al [2], CBP is often described as a persistent condition with more than one-third of patients suffering for more than 3 years and restricting the daily activities of approximately one-third of the population annually. It is one of the most common symptomatic reasons people seek health care [2]. The costs of CBP in the European Union are considerable and have been estimated to exceed €12 billion each year [3]. As one of the most common causes of disability and sick leave, there is a need to develop new and cost-effective ways to manage the condition [2,3].

### Internet-Based Interventions

One such way is through Internet-based interventions. They can play an important and compensatory role in helping CBP patients to develop appropriate self-management attitudes and strategies. In recent years, the Internet has become a prolific source for health information [4]. Today, there are hundreds, if not thousands, of health-related Internet intervention websites. In many cases, they have become a source of support for people with similar health conditions. In particular, these online programs can improve users’ knowledge and perceived social support, and can therefore have a positive impact on health attitudes and the behavior of people affected by chronic conditions [5-8].

In the last 15 years, the effectiveness of Internet-based interventions has been investigated in many different chronic conditions such as headache, panic disorder, cancer, eating disorders, and, more recently, fibromyalgia and back pain [4-10]. The results of these studies are promising and indicate that Internet-based interventions are effective in improving self-management skills and self-help capabilities, and they represent a cost-effective alternative to traditional health care services [9]. The effectiveness of Internet-based interventions is now well documented by several reviews and meta-analyses [11-17].

Despite the positive outcomes of these interventions, research has also identified some limitations of assessments of Internet-based interventions [16,17]. From a methodological point of view, some findings from previous studies have been interpreted as equivocal because they did not respond to the scientific criteria of clinical trials. Most of the studies were observational, not controlled, and carried out with specific cohorts of participants [4,9]. Other studies failed to describe randomization adequately or to blind patients to the treatment group they belonged to [4,9].

From a theoretical point of view, many studies on Internet-based interventions have tried to answer the question of whether they were effective without investigating how this effect came about [18]. This means many interventions were treated as a “black box”, without any noticeable focus on the different functions and components of their application. In order to understand how an intervention can be effective, the effective elements of interventions can be identified by appropriate experimental research. The design for that is straightforward: one group is given access to elements of which the effectiveness is to be studied while a control group is denied that access. The differences in measured effects can be clearly attributed to the elements under study.

### Interactivity in Internet-Based Interventions

In the context of Internet-based interventions, a variable to be considered for such research is one of the major features of the Internet—its potential for interactivity [19]. There are two elements that constitute interactivity. As Sundar claimed: “One of the simplest ways to conceptualize interactivity is as a feature of the *medium*, specifically the variety of modalities that it offers for the user to experience the various parts of a website, from simple text to graphics, animation, audio and video” [20]. This variety of modalities enriches the speed, the range, and the mapping of the information, which are the three defining elements of interactivity according to Steuer [21]. *Speed* refers to “the rate at which input can be assimilated into the mediated environment”, *range* refers to “the number of possibilities for action at any given time”, and *mapping* refers to “the ability of a system to map its controls to changes in the mediated environment in a natural and predictable manner” [21]. The other element of interactivity is the potential for exchange. That means users cannot only choose what they get from the intervention and how they get it, they also have a chance to talk back to the medium and be talked back to in return. That is, they can ask questions and get tailored answers, they can answer questions others ask them, they can post their experience and receive reactions, and they can react to other people’s experiences [22].

Interactivity can positively affect patient empowerment [19]. Patient empowerment is defined as a complex construct that includes different individual competencies and skills. According to Perkins and Zimmerman [23], empowerment goes beyond self-esteem, self-efficacy, competency, locus of control, and other traditional psychological constructs and can be considered a multilevel and multidimensional construct [24-26] closely linked to self-determination [27,28] and self-efficacy [29,30]. Moving from these considerations and favoring a psychological perspective, Thomas and Velthouse [31] proposed a cognitive model of empowerment, defined as increased intrinsic task motivation, where task motivation involves positively valued experiences that individuals derive directly from a task. In this respect, empowerment “can refer to feelings of power, control, and self-esteem that lead the patient to value autonomy—and thus interest in and desire to participate in health care decisions. This makes empowerment and its dimensions motivational constructs, and empowerment can be called volitional in this vein” [32,33]. Although evidence for the linkage between interactivity and patient empowerment is scarce [19,34], the former is said to enhance the latter because it helps individuals to be active, stimulates a positive attitude to learning, and enhances the value of autonomy [35-37]. Being a motivational construct, patient empowerment is considered a predictor of self-management behaviors, which ultimately affect the health of chronically ill patients [38].

Interactivity affects not only empowerment. Self-management behaviors as well as patients’ health status are also deemed to be impacted by Internet-based interventions, especially its interactive features [39-41]. If good self-managers in reality are better able to cope with CBP, this experience should be discernable in the stories and experiences related on the interactive sections of the website [39]. A person using these sections should therefore be likely to find examples of how self-management helps other patients cope with the condition. Such positive examples should lead to the conclusion that one’s own methods of self-management could be helpful in coping with the condition. This in turn should reinforce the impression that it is important what one can do to better cope and should thus, on a more general level, reinforce the impression one has of one’s own empowerment in dealing with the disease. Therefore, the interactive elements in health care websites can be expected to augment health self-management.

Other outcomes that may be influenced by Internet-based interventions, particularly the interactive sections on these interventions, include physical exercise and medication adherence [40,41]. Physical exercise is generally recommended for effectively reducing or better coping with CBP [40] and is therefore the major device for self-management and, as such, a prime target of Internet-based interventions. Medication adherence (in other words, reduced medication misuse) is equally important with respect to reducing back pain without putting one’s life at risk.

### The ONESELF Website

This study focuses on the evaluation of a specific Internet based-intervention and its interactive features called *ONESELF* [42]. The website was first implemented in 2008 to support finding information and learning how to manage CBP and, since 2009, fibromyalgia. Research has shown that the website, which is available in Italian, was by and large successful [19,34,41,43]. It was developed by the Institute of Communication and Health of the Università della Svizzera italiana (Switzerland) in collaboration with a team of rheumatologists and physiotherapists. The health team produced the medical contents and was available to interact with subscribed patients. Communication experts reframed the contents, making them comprehensible for the general public. The website was re-launched in 2013 with a completely new interface and a widening of its scope to include rheumatic arthritis.

For this study, a modified version of the original website was created, restricting access to content on CBP only. A choice of static features including the Library, the First Aid section, and a Frequently Asked Questions (FAQ) section as well as interactive features including the Virtual Gym and the Testimonials and Commentaries sections were maintained from the ONESELF website (for a detailed description, see [19,34,41,43]). In addition, two interactive features were newly developed and implemented: a weekly Action Plan and a Quiz Game. The weekly Action Plan required patients to select at the beginning of each week from a predefined list one or more physical activities of varying intensity to be completed during the week. Reminder short message service (SMS) supported patients in complying with the plan. This feature was added based on insights into its effectiveness on chronic disease management from previous online and offline interventions [44-46]. The Quiz Game was an online examination test that allowed patients to test the information learned during navigation of the website. Patients received a multiple choice question at the end of each visited section. For every correct answer, patients earned virtual points. The sum of these points was used to classify patients in a ranking that was available to all study participants of the intervention group so that patients could see how they scored in comparison to others. This form of interactivity through feedback was proposed in the context of gamification, with the aim of using game thinking and game mechanics in non-game contexts to engage users in improving knowledge on CBP and patient empowerment [47]. Screenshots of the modified ONESELF website are available in Multimedia Appendix 1.

### Study Objectives

The aim of the present randomized controlled study is to understand not only whether Internet-based interventions like ONESELF can impact patient empowerment, self-management behaviors, and, ultimately, the health status of CBP patients, but also how this can be achieved through interactive features. Thus, we propose two major hypotheses pertaining to the four desirable outcomes: patient empowerment, patients’ improvement of self-management in terms of increased physical exercise and reduced medication misuse, and lower pain burden. These outcomes will improve in CBP patients over the course of the Internet-based intervention—Hypothesis 1 (H1): there will be improvement at the time of the midterm assessment over the baseline assessment and improvement at the time of the final assessment again over the baseline assessment; and Hypothesis 2 (H2): the improvement in the desirable outcomes (empowerment and physical exercise) as well as the decrease in the undesirable outcomes (medication misuse, pain burden) will be larger for CBP patients with access to the interactive sections than for patients denied this access.

## Methods

### Study Design

**Figure 1:**
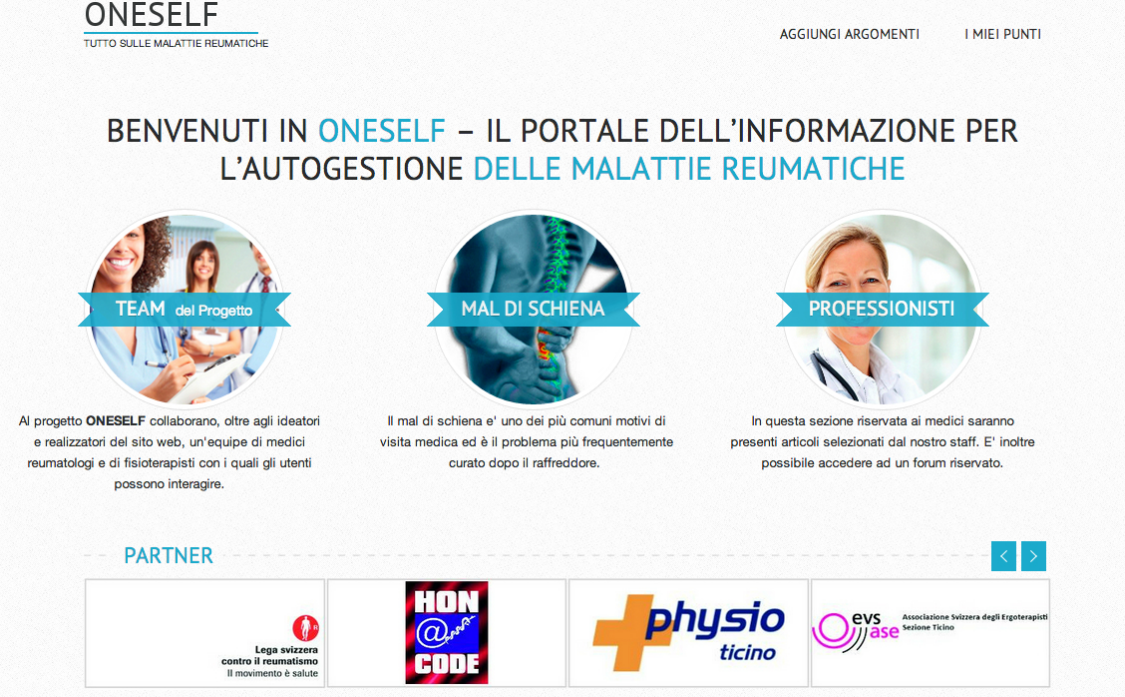
ONESELF home page.

**Figure 2:**
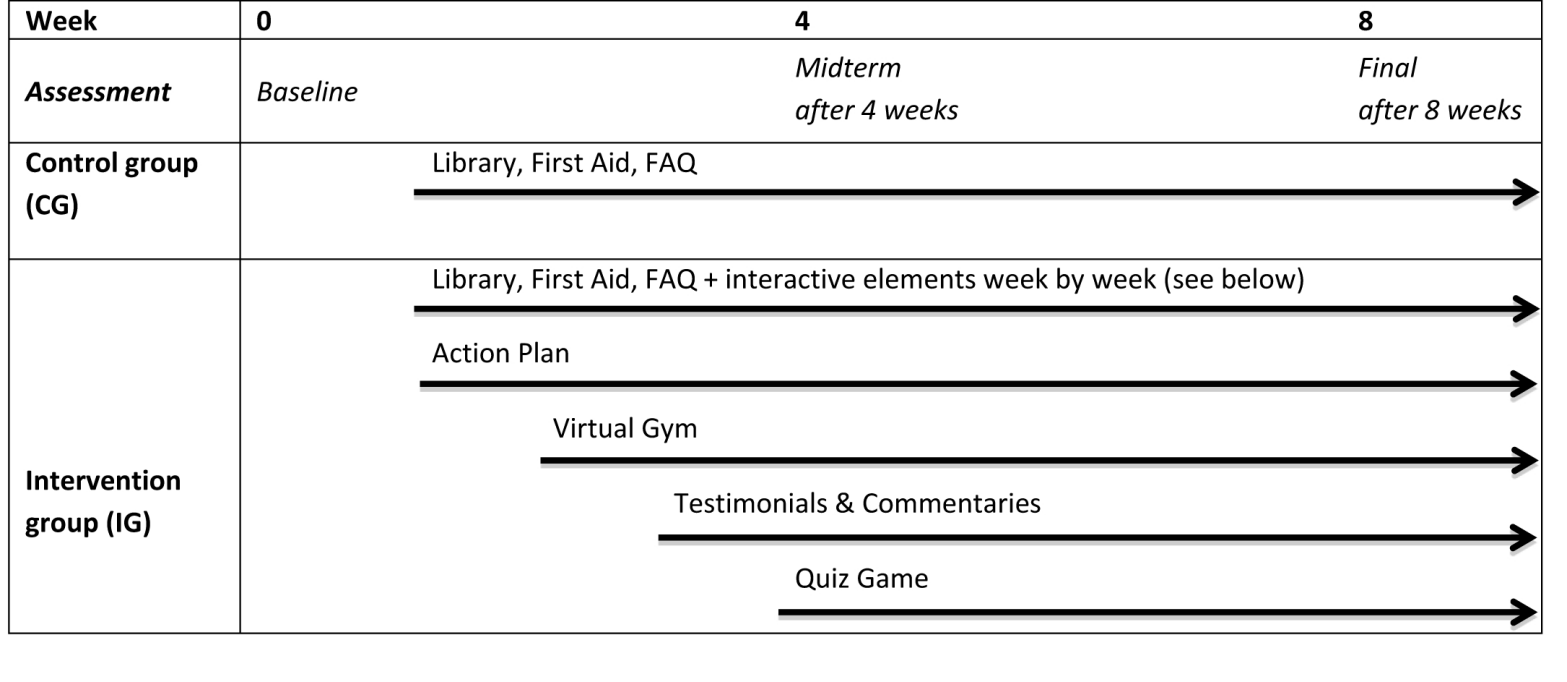
Design and timeline of randomized controlled study.

To investigate the effect of interactivity, a randomized parallel controlled study was designed (NCT02114788). Two different versions of the modified website were created, one containing only static features (ie, Library, First Aid, and FAQ) and the other containing both static and interactive features (ie, Virtual Gym, Action Plan, Testimonials and Commentaries, and Quiz Game; see Figure 1 for home page). For the intervention group, however, access to the complete version was not granted from the beginning as interactive features were added consecutively week by week as shown in Figure 2. This way, patients in the intervention group had the opportunity to become gradually familiar with the interactive features and to focus week by week on specific content and activities. Patients were blinded to the arm to which they were randomized.

### Recruitment

**Figure 3:**
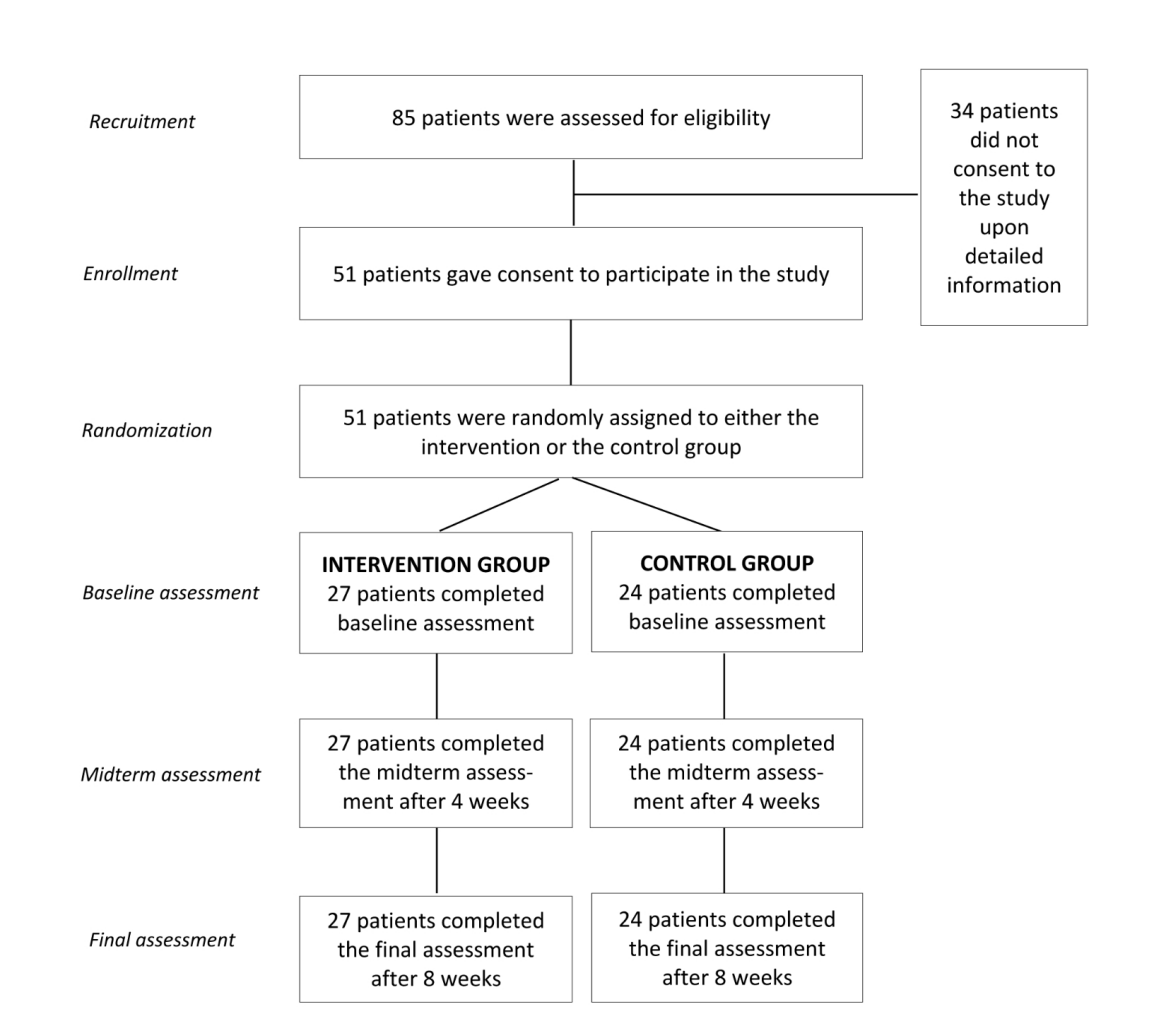
Screening, recruitment process, and random assignment.

Prospective participants were continually screened from February to June 2013 through their health care provider at selected clinics and rehabilitation centers in the Italian-speaking Canton Ticino (Switzerland). In each clinic and rehabilitation center, at least one health care provider was identified as a reference person who introduced the study to patients meeting predefined inclusion criteria. These were: (1) aged >18 years, (2) having suffered from back pain for at least 3 months, (3) no concurrent involvement in other studies, and (4) Italian native speakers. Patients who met these inclusion criteria and who showed interest in the study were asked to fill out a response card including their email address. A total of 85 response cards with valid email addresses were obtained. These patients subsequently received an email with a link to a detailed description of the study including an informed consent paragraph. Of the 85 interested patients, 51 eventually agreed to participate in the study. Figure 3 gives additional details of the screening, recruitment, and randomization process. The study, including the described recruitment procedure, was approved by the Ethics Committee of the Canton Ticino (Rif.CE 2337).

### Procedure and Randomization

The enrollment period started at the end of March 2013, and the last patient was enrolled at the end of June 2013 (3 months). Participants had access to the modified website over the course of 8 weeks. The entire study finished at the beginning of September 2013.

After confirming eligibility and obtaining informed consent from the patient (via email), the study coordinator randomly allocated participants to the two-armed parallel groups using a freely available computerized random number generator program. A permuted block randomization design method was used during the 3-month enrollment period to ensure roughly equal numbers of patients were allocated to each group. There was no face-to-face contact between the patients and research team at any point in the study, which allowed participants to live anywhere in Canton Ticino (Southern Switzerland). Of the 51 participants, 27 were allocated to the intervention group and 24 to the control group. Each participant logged in with a unique user ID so that no identifying information would be linked to their assessment, and the data were stored on secure servers. A password-protected document linking participant names to user IDs was maintained by the study coordinator, but this was not accessible to individuals involved in analyzing outcome data. Before granting access to the website, all participants were asked to complete an online questionnaire for baseline assessment. After 4 weeks, participants were asked to complete an online questionnaire for midterm assessment, and, after 8 weeks, this was repeated to get a final assessment.

### Outcome Measures

#### Overview

All outcome measures in this study were developed and validated in English. These measures were translated into Italian and adapted to the Ticino context following standardized procedures as reported in previously published studies [19,34]. For the present study, we assessed internal consistency among patients with CBP in order to confirm the reliability of the scales in our specific context.

#### Empowerment

Empowerment was measured with the Psychological Empowerment Scale [31] originally developed by Thomas and Velthouse, already cited above. The scale was originally developed for use in the workplace setting and it was adapted to be used in the health care setting [19,34]. According to the authors, empowerment is a multidimensional concept composed of four cognitive dimensions (or task assessments): (1) impact (or the degree to which behavior is seen as “making a difference”), (2) competence (or the degree to which a person can perform task activities skillfully), (3) meaningfulness (or the individual’s intrinsic caring about a given task), and (4) choice (or whether a person’s behavior is perceived as self-determined) [48]. This conceptualization aims at psychological empowerment, that is the subjective impression that one has mastery over one’s health decisions. Incorporating the multidimensionality of the concept, the scale used in this study consisted of three items adapted to the context of CBP for each of the four subdimensions: meaning (eg, “Dealing with my back pain is very important to me”), competence (eg, “I am confident about my ability to do deal with back pain”), self-determination (eg, “I have significant autonomy in determining how I deal with my back pain”), and impact (eg, “My control over the management of my back pain is large”). Participants responded on a Likert scale ranging from 1 (strongly disagree) to 7 (strongly agree) with higher values suggesting higher levels of psychological empowerment. At all three assessment points, the four subscales presented good internal consistency with an alpha value ranging from .71 to .94.

#### Medication Misuse

Medication misuse was measured with the Prescription Medication Use and Perception of Risk Instrument [49]. The scale includes six “yes/no” statements. A final sum score was obtained, which provides greater sensitivity ranging from 0 (no medication misuse) to 6 (high medication misuse) with higher scores indicating greater levels of medication misuse. Using the Kuder-Richardson-20 coefficient (KR-20) for dichotomous variables [50], psychometric testing indicated that the scale was reliable at all three assessment points (KR-20 ranging from .68 to .80).

#### Physical Exercise

Physical exercise in leisure time was measured with the respective subdimensions from the Short Questionnaire to Assess Health-Enhancing Physical Activity [51]. A sum score was calculated for the amount of time spent on physical exercise (hours) per week.

#### Pain Burden

Pain burden was measured with six items from the Chronic Pain Grading Scale [52]. Three items measured pain intensity on an 11-point scale ranging from 0 (no pain) to 10 (pain as bad as it could be). Another three items measured pain disability on an 11-point scale ranging from 0 (no interference/no change) to 10 (unable to carry on activities/extreme change). Higher values imply worse health status. At all three assessment points, the two subscales presented good internal consistency with alpha values ranging from .74 to .92 except for the pain disability scale that obtained a lower internal consistency at the final assessment (α=.62).

### Data Analysis

To estimate the general effect of the intervention, between-group differences in outcome measures were analyzed with a mixed-design analysis of variance (ANOVA). A mixed-design ANOVA is used to test for differences between two or more independent groups while subjecting participants to repeated measures. Thus, in a mixed-design ANOVA model, one factor (a fixed-effects factor) is a between-subjects variable and the other is a within-subjects variable. Thus, overall, the model is a type of mixed-effect model. Between-group effect sizes were calculated according to Cohen’s *d*. Traditionally, effect sizes of 0.20 are interpreted as “small” effects, 0.50 as “moderate” effects, and 0.80 as “large” effects [53].

Testing H1, that is the improvement in patient empowerment and physical exercise as well as the decrease in medication misuse and pain burden, the development of self-report measures over the three assessment points was looked at. Changes over time were analyzed with paired samples *t* tests. Testing H2, that is the stronger improvement in patient empowerment and physical exercise as well as the stronger decrease in medication misuse and pain burden in the intervention group over the control group, made use of the randomized controlled study design and potentially yielded strong evidence for the incremental effect of interactive features over merely static informational features. Differences between the two versions of the intervention (static vs interactive) were analyzed with independent samples *t* tests and with chi-square tests for categorical variables. Differences in change over time were not determined on the aggregate but on the individual level and then averaged. This allowed the use of *t* tests for significance testing.

Eventually, a multivariate ANOVA was conducted to examine whether the conditions differed in their use of the website and the level of satisfaction with the Internet-based intervention.

### Sample Size Determination

To achieve a power of 80% with 95% confidence to detect a clinically important difference of 1.0 point on the Psychological Empowerment Scale [31], assuming a standard deviation of 1.5 points similar to that found in other online intervention studies conducted in the context of ONESELF [19] and CBP [54-56], a minimum of 45 participants in total were required [57]. In the present study, 51 patients were enrolled to allow for dropouts. Calculations were done for a medium effect size (*d*=0.50) for group differences after the intervention.

## Results

Table 1 compares participants’ sociodemographics divided by intervention and control group. There were no significant differences for any patient characteristics, although there was a trend toward higher education among participants in the intervention group.

Table 2 shows the average scores of the outcome measures studied under the different conditions and at different assessment points. More precisely, it contains mean differences in the outcome measures within the intervention group and within the control group to test the impact of the Internet-based intervention over time (H1). It furthermore contains mean differences in the outcome measures between the intervention group and the control group to test the impact of interactive elements at single assessment points (H2). No differences occurred at baseline assessment, providing support for random assignment. Although data show that at baseline assessment the control group with no access to the interactive features of the Internet-based intervention tended to feel more empowered, but to exercise less, to be less prone to medication misuse, and to experience less pain burden than the intervention group, these differences were not significant except for medication misuse.

Mean differences in the outcome measures within the control group and within the intervention group show some improvements over time, but not throughout both assessment points as H1 holds. Within the intervention group with access to the interactive website features, overall patient empowerment as a mean score of all four dimensions increased significantly at the midterm assessment (mean difference=+0.8, *P*=.01) and remained stable at the final assessment (mean difference=+0.8, *P*=.01). Among the four dimensions, the increment was higher in self-determination (mean difference=+1.7, *P*<.001), meaning (mean difference=+1.4, *P*=.03), and competence (mean difference=+1.1, *P*=.03). In contrast, within the control group without access to interactive features, no significant improvement of overall patient empowerment was evident. Furthermore, physical exercise did not improve in either of the two conditions; quite to the contrary, it declined no matter whether participants were given or denied access to the interactive features of the website and no matter whether the development up to the midterm or to the final assessment is considered. At midterm assessment, medication misuse decreased only in the intervention group with access to the interactive elements (mean difference=−0.5, *P*=.11), while it marginally significantly increased in the control group (mean difference=+1.0, *P*=.09). Only in the intervention group did the decrease continue, even if the change was not significant (mean difference=−0.6, *P*=.11).

Eventually, at final assessment after 8 weeks from the start of the intervention, pain burden significantly decreased in both conditions (control group mean difference=−1.7, *P*<.001; intervention group mean difference=−1.5, *P*<.001). The support for H1 is therefore mixed; the hypothesis draws support only from the change in pain burden and contingent upon condition, empowerment, and medication misuse, while the deterioration of physical exercise challenges the hypothesis.

With regards to H2, results of a mixed-design ANOVA show a significant difference between the two experimental conditions (*F*_1.52_=2.83, *P*=.03, η^2^=0.30, *d*=0.55). Subsequent analyses of the comparison between the two experimental conditions at midterm and at final assessment indicate that the addition of interactive features very clearly improved patients’ overall empowerment. However, the majority of the differences in change from baseline assessment were significant and greater at midterm assessment (mean difference=+1.2, *P*=.03, *d*=0.63), but marginally significant and smaller at final assessment (mean difference=+0.8, *P*=.09, *d*=0.44). This is also evident considering the four dimensions of empowerment separately (especially the subdimensions meaning and self-determination). No significant difference was evident for physical exercise both at midterm and final assessment, indicating that interactivity had no incremental effect and was unable to work against the decline in exercising. For medication misuse, the differences were as expected and highly significant, meaning that interactivity clearly helped to curb this deteriorating behavior both at midterm assessment (mean difference=−1.5, *P*=.04, *d*=0.28) and final assessment (mean difference=−1.6, *P*=.03, *d*=−0.55). Eventually, interactivity had no significant effect on decrease of the burden caused by CBP. Thus, H2 receives strong support from looking at the outcomes of empowerment and medication misuse, but no support from looking at physical exercise and pain burden. There is, however, no outcome that runs against the hypothesis.

Eventually, a multivariate ANOVA was conducted to examine whether the conditions differed in their active participation in the intervention. Both the intervention and the control group were compared with regard to the use of the website, its evaluation as a means to improve CBP, and the frequency of navigation, which is the number of visited pages per week (Table 3).

Inspection of the univariate tests indicated that the difference between the two experimental conditions was significant for the frequency of navigation (*P*=.01), the evaluation of the website for improvement of CBP (*P*<.001), and the number of visited pages (*P*<.001). Participants in the intervention group, on average, used the website more often and considered it more effective for improving CBP than participants in the control group. The sections most visited by participants in the intervention group were the Library (48%, 13/27) and the Virtual Gym (33%, 9/27), while the sections more visited by the control group were the Library (80%, 19/24) and FAQ (12%, 3/24). Between-group comparison at both assessment points showed that the intervention group used the website more often and evaluated it as more beneficial. Furthermore, within- group comparison showed that in both the intervention and the control group website use significantly decreased over time showing a “wearout effect”, while the evaluation of the website for CBP improvement remained stable.

**Table 1:** Sociodemographic characteristics of participants at baseline assessment.

Characteristic		Control group, (n=24) n (%)	Intervention group, (n=27) n (%)	Significance
**Gender**				0.89^a^
	Female	12 (50.0)	14 (51.9)	
	Male	12 (50.0)	13 (48.1)	
**Highest educational attainment**				0.13^b^
	Primary school	1 (4.1)	1 (3.7)	
	Secondary school	7 (29.1)	3 (11.1)	
	High school	13 (54.1)	14 (51.9)	
	University	3 (12.7)	9 (33.3)	
**Currently in professional occupation**				0.81^b^
	Yes	14 (58.3)	16 (59.3)	
	No	10 (41.7)	11 (40.7)	
Age, mean (SD)		51 (14.1)	44 (13.6)	0.58^b^
Pain duration in years, mean (SD)		9.3 (8.7)	7.9 (7.2)	0.64^b^

^a^Chi-square test

^b^Independent samples *t* test

**Table 2:** Means, mean differences, and significance levels for outcome measures within and between two experimental groups.

		Baseline assessment (BA)			Midterm assessment (MA)				Final assessment (FA)				Difference in change from BA to		Difference between BA and MA		Difference between BA and FA	
		CG^a^	IG^b^	Diff	CG	IG	Diff	d^c^	CG	IG	Diff	d	MA	FA	CG	IG	CG	IG
**Hypothesized difference**				±0			+				+		+	+	(+)	+	(+)	+
	Empowerment: total score	4.5	4.0	−0.5	4.1	4.8	+0.7^d^	0.63	4.5	4.8	+0.3	0.44	+1.2^e^	+0.8^d^	−0.4	+0.8^e^	+0.05	+0.8^f^
	Empowerment: meaning	4.9	4.4	−0.5	4.4	5.3	+0.9^e^	0.70	5.2	5.3	+0.1	0.09	+1.4^e^	+0.6	−0.5	+0.9^d^	+0.3	+0.9^e^
	Empowerment: competence	4.5	4.0	−0.5	4.0	4.6	+0.6^d^	0.53	4.5	5.0	+0.5	−0.35	+1.1^e^	+0.9^d^	−0.5	+0.7^d^	0.0	+1.0^g^
	Empowerment: self-determination	4.4	3.7	−0.7^d^	3.9	4.9	+1.0^e^	0.71	4.2	4.6	+0.4	0.27	+1.7^g^	+1.1^d^	−0.5	+1.2^g^	−0.2	+0.9^f^
	Empowerment: impact	4.3	3.8	−0.5	4.2	4.6	+0.4	0.34	4.2	4.3	+0.1	0.16	+0.9	+0.6	−0.1	+0.8^d^	−0.1	+0.5
	Physical exercise	1.4	2.2	+0.8	0.7	1.4	+0.7	0.36	0.3	1.3	+1.0^d^	−0.48	−0.1	+0.2	−0.7	−0.9	−1.1^e^	−0.9
**Hypothesized difference**				±0			−				−		−	−	(−)	−	(−)	−
	Medication misuse	0.8	1.9	+1.1^e^	1.8	1.4	−0.4	0.28	2.0	1.3	−0.6	−0.55	−2.5^g^	−1.6^f^	+1.0^d^	−0.5	+1.2	−0.6
	Pain burden	3.8	4.3	+0.5	3.0	3.9	+0.9^d^	0.48	2.1	2.8	+0.7	0.49	+0.4	+0.2	−0.9^d^	−0.4	−1.7^g^	−1.5^g^

^a^CG: control group

^b^IG: intervention group

^c^ *d*=between-group effect sizes according to Cohen’s *d*; independent samples *t* test between CG and IG for each assessment point and for the differences in change from BA to MA and BA to FA; paired samples *t* test for differences between assessment points for control and intervention group.

^d^ *P*<.10

^e^ *P*<.05

^f^ *P*<.01

^g^ *P*<.001

**Table 3:** Means and mean differences for use and evaluation of the website between two experimental groups.

Use/Evaluation	Midterm assessment (MA)			Final assessment (FA)			Difference between FA and MA	
	CG^a^	IG^b^	Difference	CG	IG	Difference	CG	IG
In the last four weeks, how often did you navigate the website of the study about back pain?	1.9	2.7	+0.8^c^	1.6	2.4	+0.8^c^	−0.3	−0.3
How much has the website contributed to the improvement of your back pain in everyday life?	2.5	4.0	+1.5^e^	2.4	4.0	+1.6^e^	−0.1	0.0
Number of pages visited per week	4.0	7.0	+3.0^e^	2.8	5.0	+2.2^e^	−1.2^c^	−2.0^e^

^a^CG: control group

^b^IG: intervention group

^c^ *P*<.05

^d^ *P*<.01

^e^ *P*<.001

## Discussion

### Principal Findings

Considering one of its main objectives, that is the understanding of the impact of Internet-based interventions like ONESELF on patient empowerment, the study found a moderate differential effect for the two experimental conditions. Among patients without access to the interactive sections, empowerment remained constant after 8 weeks while it significantly increased and remained consistently higher among patients who had access to the interactive sections. This suggests that the interactive sections of health care websites might indeed play a role in empowering patients with chronic conditions and gives useful insights compared to studies with contradictory results that did not pay attention to the presence or absence of interactive website features. Further evidence for the empowering effect of interactive features could be gained by looking at the actual use of these, as we would expect heavy users of interactive features to demonstrate a larger increase in empowerment than light users of these features. Future studies are needed to test this hypothesis. The differential effects of the website versions on patient empowerment refer to an overall score across all four dimensions of psychological empowerment. But they hold for each of the four dimensions too. This suggests—beyond the analyses of the psychometric qualities of this scale—that the four dimensions indeed belong together and contribute to the overall concept of empowerment. Considering the four dimensions separately, patients in the intervention group significantly improved their perceived self-determination, meaningfulness, and competence.

With regard to the differential effect of the website versions on self-management behaviors related to CBP, the results of this study show a considerable decline of physical exercise at both the midterm and the final assessment, irrespective of the experimental condition. One explanation could be that the use of websites like ONESELF, independent of the presence of interactive features, prevents people from doing what is good for them, in this case exercising to relieve pain. This, however, would run against the explicit objectives and contents of the Internet-based intervention, which put great emphasis on the necessity of exercising (the website used weekly action plans with reminder SMS messages aimed at motivating CBP patients to engage in regular physical exercise), and it would also run against the findings of other studies [58-60]. Other more probable explanations for the lack of impact on physical exercise could be a wearout and a measurement effect linked to the Internet-based intervention. The wearout effect describes the decrease in website use between the midterm and the final assessment with impact on the overall effectiveness of the intervention at final assessment. The measurement effects describes seasonal effects related to the period of enrollment since almost half of the participants (43%, 22/51) reported on their physical exercise in July and August, which are both popular holiday months in Switzerland where many people interrupt their habitual activities including physical exercise.

Results show that, overall, medication misuse did not change much as a result of the Internet-based intervention. That, however, hides very different developments in the two experimental groups: while misuse went up in the control group, it went down in the group with access to the interactive features, even though the difference between midterm and final assessment is not significant. Increased medication misuse as a consequence of a health care website is difficult to interpret but cannot be completely ruled out. No matter where the increased misuse in the control group may originate from, interactivity appears to have the potential to work against that, at least in keeping control over the use of such medications and adhering to medical regimes.

Eventually, participants experienced less pain as the exposure to the Internet-based intervention proceeded. If the intervention contributed to this decline, it was not due to its interactive features as the decrease in pain burden was observed in both groups. Strangely enough, we observed over the course of the experiment a reduction in physical exercise but a clear improvement of the pain condition. The most straightforward interpretation of this aggregate result would be that, contrary to most assumptions, the relationship between exercise and pain is different than expected. But to posit a positive relationship—more exercise, more pain—would certainly be premature, if for the fact alone that the increased misuse of medication among the control group would be difficult to explain. However, both developments could be again explained by seasonal effects. Measurement in summer might be responsible for both low levels of physical exercise due to a break of habitual behaviors for holidays, and lower levels of pain than in other times of the year with cold and rainy weather. Moreover, a lower level of back pain might be ascribed to a diminishment of work and work-related stress that can contribute to a decreased level of pain [61-64].

Back pain patients with access to the static elements of ONESELF providing information only gave up on their exercise, felt less pain, and reported more medication misuse. Participants of the intervention group with access to the interactive elements on top of the informative ones also gave up on their exercise, also felt less pain, but reported less medication misuse. These patients felt more empowered through the Internet-based intervention as compared to patients of the control group, and they reported to have better mastery over their CBP at the end of the intervention. This result was also confirmed by significant differences between the intervention group and the control group in the evaluation of the intervention as an effective means to contribute to the improvement of CBP in everyday life.

We can, therefore, conclude that the interactive features of the ONESELF website indeed contributed to improving patient empowerment while purely static elements with information only did not. Hence, this study complements the emerging literature supporting the utility of Internet-based interventions aimed at patient empowerment. The empowered patient emerges as a person who does not passively receive information, but takes increased responsibility for and a more active role in decision-making regarding his or her health [27-30]. This study highlights how empowerment is strengthened by interactivity, and this result enhances the existing literature in the field about the conjunction of these two constructs [35-37].

### Limitations

This randomized controlled study is not without any limitations, which are mainly of a methodological nature. First, the study suffers from a small group size, despite the significant differences found between the two conditions. A bigger sample size might have strengthened marginally significant results and helped to detect significant differences within the intervention group for physical exercise. Second, the study lacks a pure control group. In fact, patients provided with the static version of the website were used as a control group, but no group of CBP patients was included with no access to the Internet-based intervention at all. However, the main objective of this study was to test the effectiveness of interactive sections compared to static elements only and not to test the effectiveness of the intervention as a whole. Third, a 2-month intervention might be too short a period to discover meaningful effects and conclusions on the effectiveness of Internet-based interventions on maintaining high levels of empowerment and beneficial self-management behaviors. Finally, the present study lacks specificity inasmuch as it did not take into account the quality of any of the sections that might have caused the differences between the intervention group with interactive sections and the control group with static elements only. Further insights on which specific elements cause change are essential to better inform the design of future Internet-based interventions aimed at improving chronically ill patients’ empowerment, self-management behaviors, and, ultimately, their health status.

### Conclusions

In conclusion, this randomized controlled study provides evidence that interactive features of Internet-based interventions aimed at chronic pain management appear mostly to affect soft outcomes related to self-perception including patient empowerment and pain representations, while the harder behavioral outcomes such as physical exercise seem to be unaffected. Nevertheless, this study adds to the growing body of literature demonstrating the effectiveness of Internet-based interventions on the management of chronic diseases like CBP. As the Internet increasingly becomes a major source of medical information and social support, this study demonstrates that it can also be an efficient and effective tool for patient empowerment that—together with health knowledge—is considered an important predictor of constructive self-management behaviors and positive health outcomes.

## Multimedia Appendix 1

Screenshots of ONESELF.

## Multimedia Appendix 2

CONSORT-EHEALTH checklist V1.6.2 [65].

## Abbreviations

ANOVA: analysis of variance
CBP: chronic back pain
SMS: short message service
