# Supplementary material for: Interactive Sections of an Internet-Based Intervention Increase Empowerment of Chronic Back Pain Patients: Randomized Controlled Trial
Source: J Med Internet Res. 2014 Aug 13;16(8):e180. doi: 10.2196/jmir.3474 (PMC4147711; doi:10.2196/jmir.3474)

ONESELF hompage

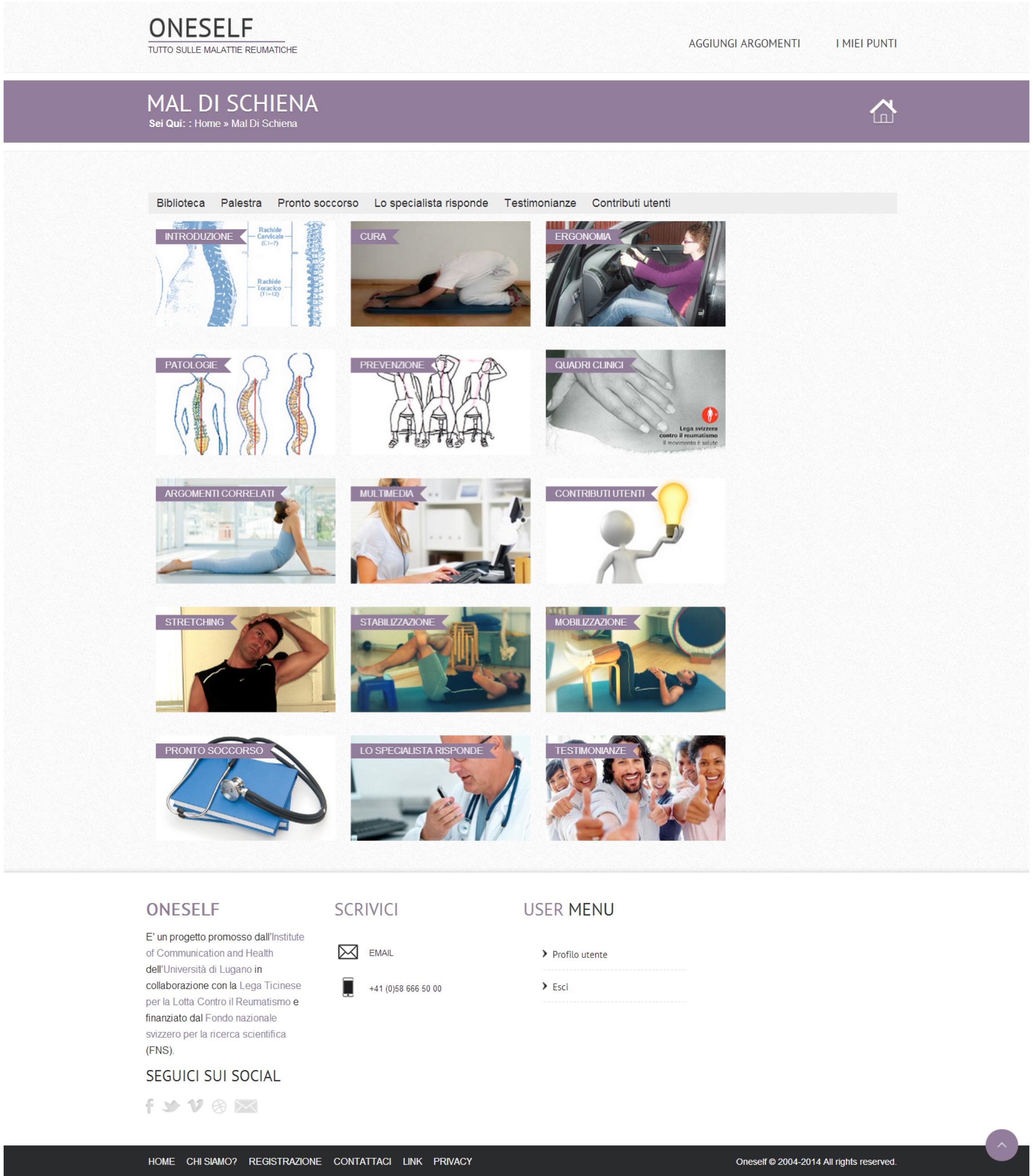

Library

MAG 15, 2013

I medicamenti

I medicamenti non guariscono il mal di schiena, ma aiutano ad alleviare il dolore. Di seguito vengono presentati alcuni medicamenti, tra i più diffusi, che sono utilizzabili sia nel trattamento del mal di schiena acuto che cronico.

Prima di iniziare qualunque trattamento, è bene assicurarsi che il proprio medico l'approvi.

- Il paracetamolo può essere acquistato senza ricetta medica. E' un farmaco che toglie il dolore piuttosto rapidamente e risparmia lo stomaco. Vista la buona tollerabilità, va provato in ogni caso. In alcuni casi di dolore particolarmente acuto, il paracetamolo non è sufficiente.
- AINS (Antinfiammatori non steroidali), si tratta di una famiglia di numerosi farmaci privi di cortisone. Molti AINS possono essere ottenuti con ricetta medica. Essi hanno un'influenza sulle patologie infiammatorie e, in particolare, a livello periarticolare e periradicale. Hanno un'azione antidolorifica sulla muscolatura che viene potenziata in associazione con i mirorilassanti.
- I farmaci antidolorifici con azione a livello centrale derivano dagli oppiacei. Essi sopprimono i segnali di dolore nel sistema nervoso centrale, agendo sul centro di sensibilità al dolore nel cervello. La maggior parte di questi farmaci possono essere ottenuti solo con ricetta.
- I miorilassanti sono farmaci che aiutano ad alleviare il dolore rilassando la muscolatura. Di solito, vengono prescritti in combinazione con AINS e analgesici.
- Gli antidepressivi aiutano a ridurre lo stress emozionale che talvolta accompagna il mal di schiena e [illegible]

BIBLIOTECA

- Introduzione
- Cura
- Ergonomia
- Patologie
- Prevenzione
- Quadri Clinici
- Argomenti Correlati
- Multimedia

UNA DOMANDA DEL GIOCO A [illegible]

Frequently asked questions

ONESELF
TUTTO SULLE MALATTIE REUMATICHE

AGGIUNGI ARGOMENTI | I MIEI PUNTI

I CONTRIBUTI DEI MEDICI

Sei Qui: : Home » Mal Di Schiena » Lo Specialista Risponde » I Contributi Dei Medici

DOMANDA 1

09 MAR | 0

Da: admin I contributi dei medici

Che cos'è l'auto-mobilizzazione?
Sono delle tecniche di medicina manuale.Si tratta di esercizi e movimenti che possono essere insegnati da parte di un fisioterapista e che permettono al paziente di mobilizzare in modo autonomo i vari segmenti della colonna vertebrale, come per esempio la colonna cervicale, toracale o lombare ed anche le articolazioni sacroiliache od altre articolazioni periferiche.

Leggi tutto →

DOMANDA 2

09 MAR | 0

Da: admin I contributi dei medici

Si potrebbe approfondire la relazione sport e mal di schiena?
Tenendo presente il messaggio inviato nel FORUM dall'utente villa, alcune sono le osservazioni da fare: praticare sport simmetrici rimane una indicazione di massima per chi soffre di mal di schiena cronico ma come spesso avviene in campo medico, ogni singola situazione clinica va considerata nella sua unicità e peculiarità.

Leggi tutto →

DOMANDA 3

09 MAR | 0

Da: admin I contributi dei medici

Dottore, da qualche mese soffro di un dolore a livello della regione inguinale. Potrebbe essere un'artrosi dell'anca?

"Questi sintomi sono compatibili con una coxatrosi, tuttavia la diagnosi va confermata con un approfondito esame clinico ed accertamenti radiologici. Anche disturbi alla schiena possono irradiarsi nella zona inguinale o nella coscia sia anteriormente che posteriormente."

Segnalato da Dr. Marti

Leggi tutto →

DOMANDA 4

09 MAR | 0

Da: admin I contributi dei medici

Dottore, ho mal di schiena ed un'ernia del disco trovata mediante risonanza magnetica. Devo essere operato? Di regola no. Nel 30 % delle persone sane, senza disturbi alla schiena, si possono trovare delle ernie del disco tramite risonanza magnetica e nel 50% delle persone sane delle protrusioni discali.

Leggi tutto →

DOMANDA 5

09 MAR | 0

Da: Dr. keller I contributi dei medici

Dottore cosa ne pensa del trattamento del mal di schiena con le terapie "alternative"?
"Pur non essendo un esperto di medicina alternativa provo a dare una risposta semplice a questo quesito, che sicuramente interesserà molti pazienti. L'efficacia di tutte le terapie cosiddette alternative non è ben quantificabile come invece lo richiede la medicina tradizionale, motivo per il quale è estremamente difficile.....

Leggi tutto →

LO SPECIALISTA RISPONDE

- I contributi dei medici

ONESELF

E' un progetto promosso dall'Institute of Communication and Health dell'Università di Lugano in collaborazione con la Lega Ticinese per la Lotta Contro il Reumatismo e finanziato dal Fondo nazionale svizzero per la ricerca scientifica (FNS).

SEGUICI SUI SOCIAL

SCRIVICI

EMAIL
+41 (0)58 666 50 00

USER MENU

- Profilo utente
- Esci

HOME | CHI SIAMO? | REGISTRAZIONE | CONTATTACI | LINK | PRIVACY

Oneself © 2004-2014 All rights reserved.

First aid

PRONTO SOCCORSO

MAR 23, 2013

A cura del Dr. med. Guido Mariotti

> Ti sei svegliato questa mattina e non riesci più ad alzarti dal letto a causa di un forte mal di schiena?
> Hai fatto un movimento falso con la colonna vertebrale e sei rimasto bloccato?
> Hai sollevato in modo poco ergonomico un peso e ti è venuta una forte fitta alla schiena con dolori irradianti al gluteo fino a metà coscia?
> Da alcuni giorni sentivi dei leggeri dolori alla colonna lombare che andavano e venivano e che oggi si sono intensificati e non se ne vanno più e devi camminare tutto storto e non puoi sederti?

Non ti preoccupare. Si tratta di una lombaggine acuta (il famoso colpo della strega). Il medico la definisce anche Sindrome Lombovertebrale Acuta (SLA).
Nella maggior parte dei casi si tratta di un'affezione benigna della colonna vertebrale di origine meccanica che si manifesta a seguito di alterazioni degenerative dei dischi intervertebrali o su disturbi funzionali o degenerativi delle faccette articolari accompagnata da un aumento del tono muscolare a livello della muscolatura lombo-sacrale.

L'evoluzione dei dolori e del bloccaggio è quasi sempre favorevole spontaneamente senza particolari interventi terapeutici.
Di solito i dolori durano qualche giorno (al massimo alcune settimane) tendono a diminuire di intensità e a scomparire completamente.

> Quali sono gli accorgimenti da intraprendere per aiutare questa evoluzione spontanea favorevole?

Cercati una posizione in cui i dolori sono sotto controllo e sopportabili. Nella maggior parte dei casi la posizione migliore è quella sdraiata. Cerca comunque di cambiare posizione e di muoverti un po', eventualmente camminando per brevi tratti. Nell'ambito del possibile e con le limitazioni date dal dolore cerca di riprendere il più rapidamente la tua vita quotidiana e lavorativa. Un periodo troppo prolungato (superiore ai due giorni) di riposo a letto è controproducente e prolunga la durata del dolore e il periodo di ripresa dell'attività professionale.

Localmente puoi applicare a livello della colonna lombare una borsa d'acqua fredda. Il freddo aiuta nelle prime ore e riduce il dolore.
In seguito potrebbe essere d'aiuto un impacco tiepido o caldo. Per esempio una borsa d'acqua calda, un cuscino elettrico e anche un bagno caldo o una doccia calda. Il caldo riduce il tono muscolare e di conseguenza anche il dolore.

Un massaggio alla muscolatura contribuisce a diminuire il tono muscolare. Puoi utilizzare per il massaggio una crema contenente dei medicamenti antinfiammatori. Se non l'hai a disposizione va bene anche una semplice crema per il corpo.

Vi sono poi dei semplici esercizi di ginnastica che permettono una controllata mobilizzazione dei segmenti di movimento lombari e che riducono il tono muscolare. Si tratta di esercizi chiamati di mobilizzazione senza carico della colonna lombare . Li puoi trovare descritti e dimostrati nella nostra palestra.

Per quanto riguarda i medicamenti spesso basta aiutarsi con del Paracetamolo (Panadol, Dafalgan, Zolben).
E`un medicamento efficace e con pochi effetti collaterali. Da non utilizzare se si è allergici a questo medicamento.
Il dosaggio massimo è di tre volte un grammo al giorno.
Gli Anti Infiammatori Non Steroidali (AINS) sono pure efficaci nella terapia del mal di schiena.
Sono dei medicamenti con effetti collaterali più importanti e pericolosi rispetto al Paracetamolo e vanno presi con cautela e per breve durata (al massimo 1 settimana). Se non si è mai preso un medicamento di questo tipo é meglio desistere e prima consultare il proprio medico. Gli effetti collaterali più frequenti sono le allergie, il bruciore di stomaco, la diarrea o l'ostipazione. Se presi per un periodo prolungato o a dosaggi non corretti possono provocare gravi complicazioni come l'ulcera gastrica, l'insufficienza renale e complicazioni cardiovascolari.

> Quando consultare il proprio medico?

- Se i dolori non regrediscono dopo 3-4 giorni malgrado le terapie sopra indicate.

- Immediatamente se i dolori lombari hanno un carattere particolare:

PRONTO SOCCORSO

› Domanda frequente

Action plan (only for intervention group)

PIANO D'ATTIVITA DI SILVIA HELPERTEST

Sei Qui: : Home » PIANO D'ATTIVITA Di Silvia Helpertest

Fare yoga una volta alla settimana per 30 minuti.

lunedì

Scelga il giorno della sua attività

Fare un giro in bicicletta una volta alla settimana per 30 minuti.

lunedì

Scelga il giorno della sua attività

INVIA

ONESELF

E' un progetto promosso dall'Institute of Communication and Health dell'Università di Lugano in collaborazione con la Lega Ticinese per la Lotta Contro il Reumatismo e finanziato dal Fondo nazionale svizzero per la ricerca scientifica

SCRIVICI

EMAIL

+41 (0)58 666 50 00

USER MENU

- Profilo utente
- Esci

Action plan 2

PIANO D'ATTIVITA DI SILVIA HELPERTEST

Sei Qui: : Home » PIANO D'ATTIVITA Di Silvia Helpertest

Nel Corso delle prossime 4 settimane, le sarà chiesto di creare il suo piano d'attività per ogni ciclo di 7 giorni iniziando dal giorno della registrazione. Questo significa che all'inizio di ogni settimo giorno del ciclo lei sceglierà una attività dalla seguente lista e dopo deciderà quando svolgere tale attività. La prego di scegliere la sua prima attività

- [ ] Fare attività aerobica una volta alla settimana per 30 minuti.
- [ ] Fare qualche esercizio di allungamento ogni giorno per 10 minuti.
- [ ] Fare un allenamento speciale di allungamento muscolare una volta alla settimana per 30 minuti.
- [ ] Fare nuoto una volta alla settimana per 30 minuti.
- [ ] Fare una passeggiata una volta alla settimana per 60 minuti.
- [ ] Fare yoga una volta alla settimana per 30 minuti.
- [ ] Fare un giro in bicicletta una volta alla settimana per 30 minuti.
- [ ] Indossare ogni giorno solo scarpe confortevoli.

Virtual gym (for intervention group only)

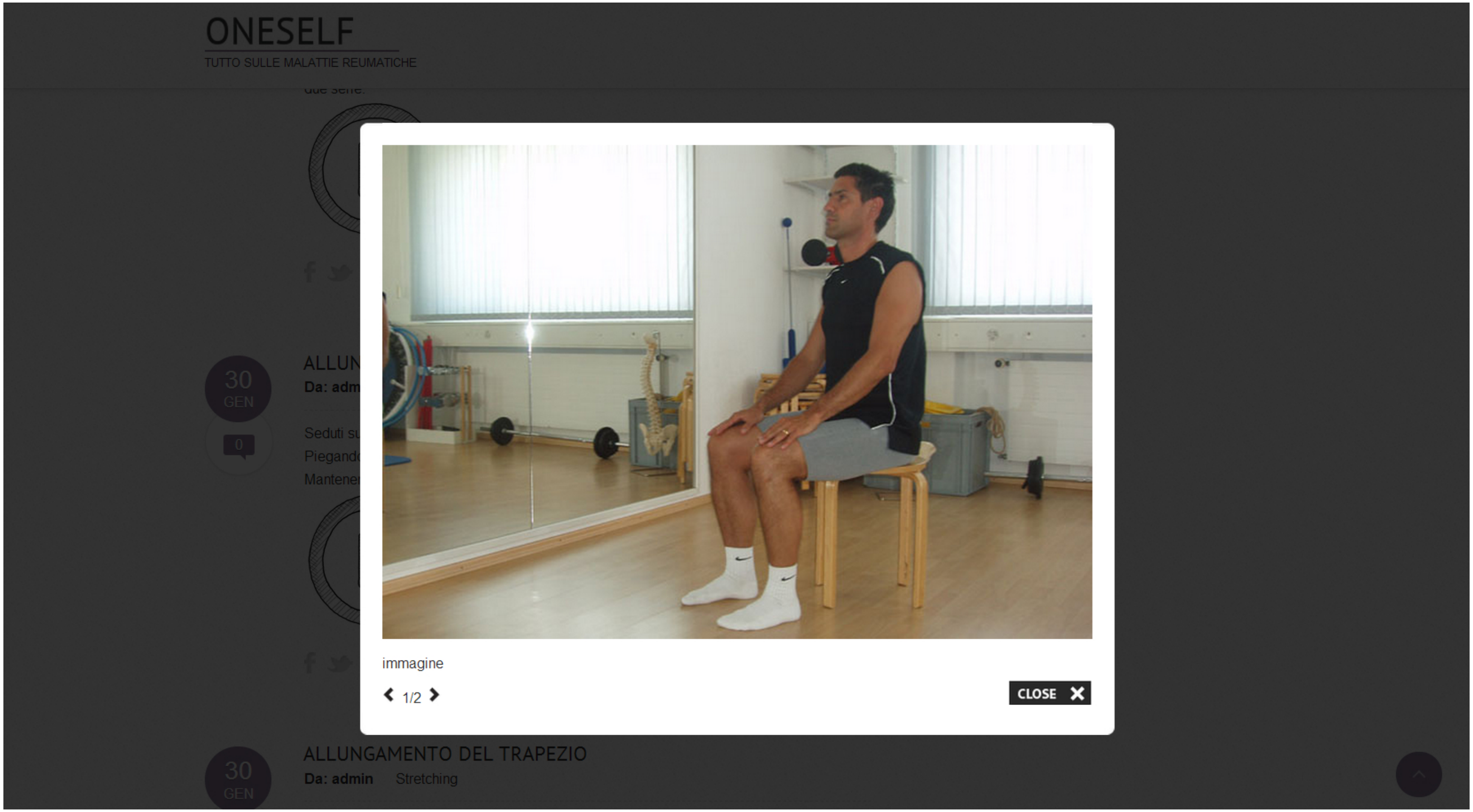

Virtual gym 2 (for intervention group only)

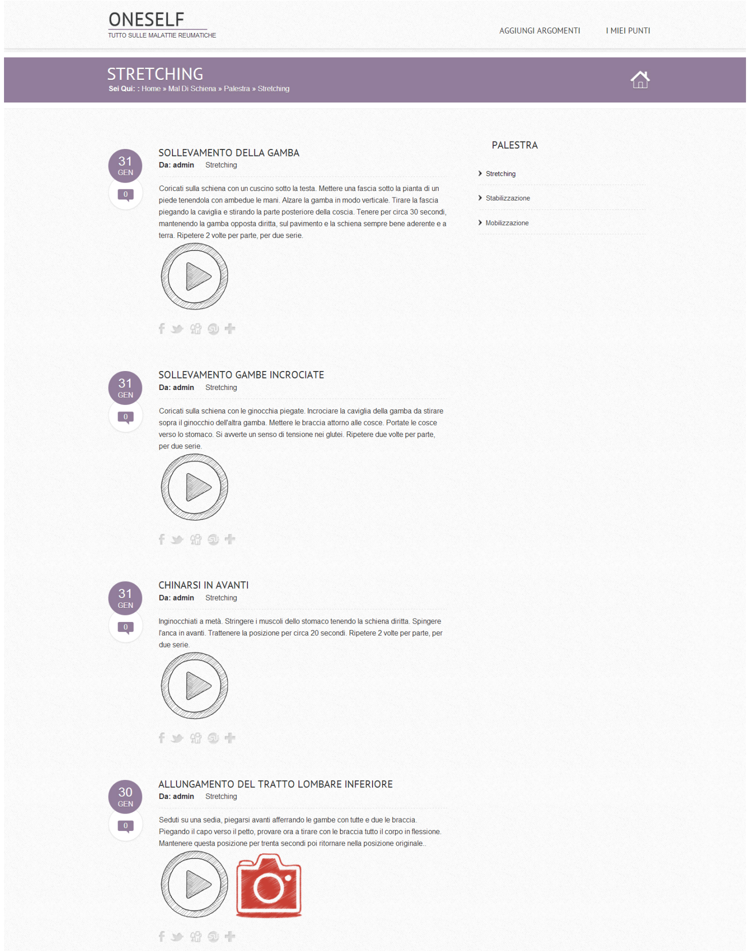

Quiz game (for intervention group only)

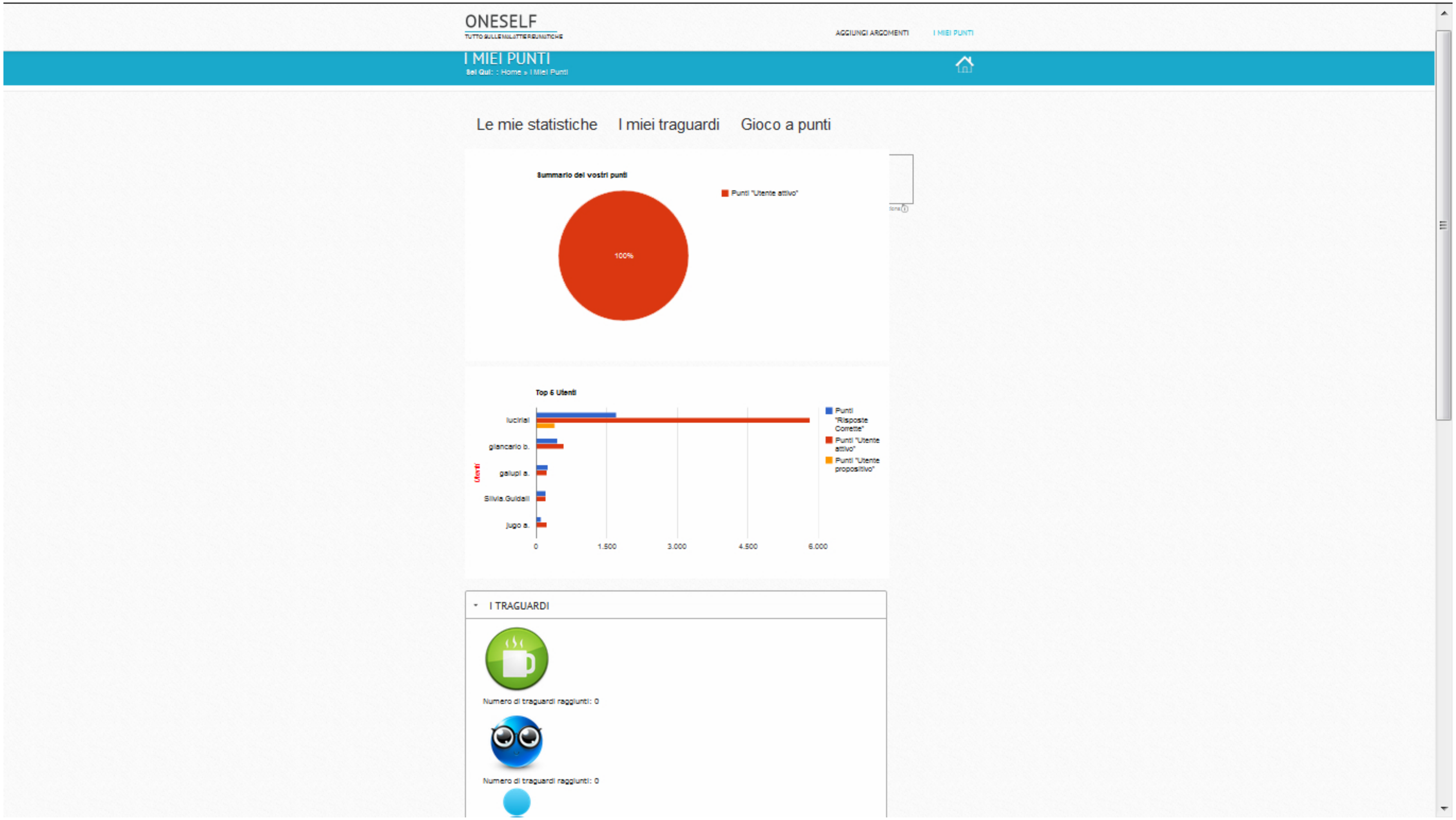

Testimonials and commentaries (for intervention group only)

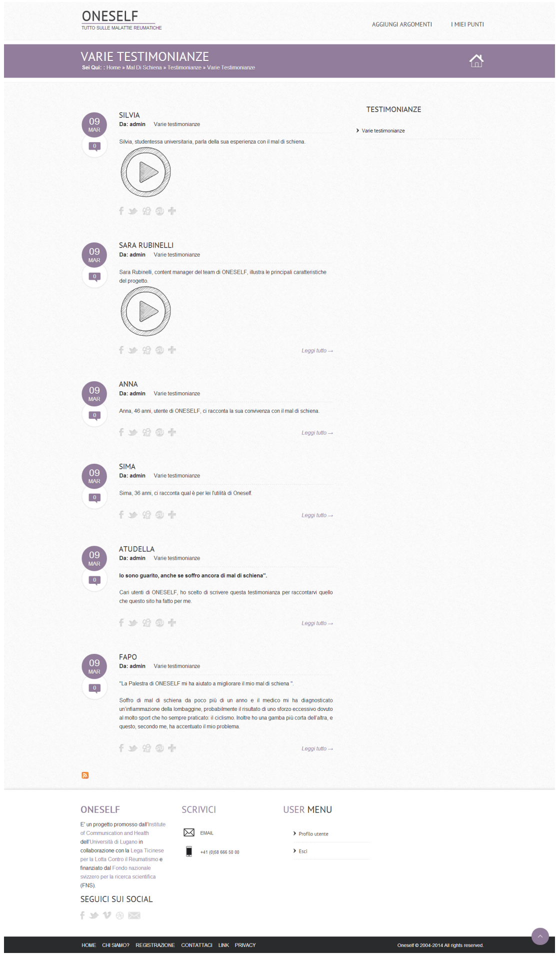

Supplement: Supplementary file 1 [file jmir_v16i8e180_app1.pdf]
