# Supplementary material for: Interactive Sections of an Internet-Based Intervention Increase Empowerment of Chronic Back Pain Patients: Randomized Controlled Trial
Source: J Med Internet Res. 2014 Aug 13;16(8):e180. doi: 10.2196/jmir.3474 (PMC4147711; doi:10.2196/jmir.3474)
Supplement: Supplementary file 2 [file jmir_v16i8e180_app2.pdf]

CONSORT-EHEALTH Checklist V1.6.2 Report

(based on CONSORT-EHEALTH V1.6), available at [http://tinyurl.com/consort-ehealth-v1-6].

3474

Date completed

4/16/2014 8:01:19

by

Silvia

Interactive sections of an internet-based intervention increase patient empowerment: a randomized controlled study with chronic back pain patients

TITLE

1a-i) Identify the mode of delivery in the title

"Interactive sections of an internet-based intervention increase patient empowerment"

1a-ii) Non-web-based components or important co-interventions in title

1a-iii) Primary condition or target group in the title

"a randomized controlled study with chronic back pain patient"

ABSTRACT

1b-i) Key features/functionalities/components of the intervention and comparator in the METHODS section of the ABSTRACT

"Fifty-one patients were recruited through their healthcare providers and randomly assigned to either an experimental group with full access to the Internet-based intervention or a control group who was denied access to the interactive sections and knew nothing thereof. The intervention took eight weeks. A baseline, a mid-term after four weeks, and a final assessment after eight weeks measured patient empowerment, physical exercise, medication misuse, and pain burden."

1b-ii) Level of human involvement in the METHODS section of the ABSTRACT

1b-iii) Open vs. closed, web-based (self-assessment) vs. face-to-face assessments in the METHODS section of the ABSTRACT

1b-iv) RESULTS section in abstract must contain use data

"All patients completed the study. Compared to the control group, the availability of interactive sections significantly increased patient empowerment and reduced medication misuse in the intervention group. Both the frequency of physical exercise and pain burden decreased, but to equal measures in both groups"

1b-v) CONCLUSIONS/DISCUSSION in abstract for negative trials

INTRODUCTION

2a-i) Problem and the type of system/solution

"Chronic back pain (cBP) is one of the most highly prevalent medical conditions and represents a significant public health problem.
The costs of cBP in the EU are very considerable and have been estimated to exceed €12 billion each year [3]. Being one of the most common causes of disability and sick leave, there is a need to develop new and cost-effective ways to manage the condition [2-3].
One such way are Internet-based interventions. They can play an important and compensatory role in helping cBP patients to develop appropriate self-management attitudes and strategies".

2a-ii) Scientific background, rationale: What is known about the (type of) system

"Despite positive outcomes of these [internet-based] interventions, research has also identified some limitations of assessments of Internet-based interventions [16-17]. From a methodological point of view, some findings from previous studies have been interpreted as equivocal because they did not respond to the scientific criteria of clinical trials. Most of the studies were observational, not controlled and carried out with specific cohorts of participants [4,9]. Other studies failed to describe randomization adequately or to blind patients to the treatment group they belonged to [4,9].

METHODS

3a) CONSORT: Description of trial design (such as parallel, factorial) including allocation ratio

"The aim of the present randomized controlled study is to understand not only whether Internet-based interventions like ONESELF can impact patient empowerment, self-management behaviors and, ultimately, the health status of cBP patients, but also how this can be achieved through interactive features. Thus, we propose two major hypotheses pertaining to the four desirable outcomes: patient empowerment, patients' improvement of self-management in terms of increased physical exercise and reduced medication misuse, and lower pain burden. These outcomes will improve in cBP patients over the course of the Internet-based intervention, that is:
H1: There will be improvement at the time of the midterm assessment (MA) over the baseline assessment (BA) and improvement at the time of the final assessment (FA) again over the baseline assessment (BA).
H2: The improvement in the desirable outcomes (empowerment and physical exercise) as well as the decrease in the undesirable outcomes (medication misuse, pain burden) will be larger for cBP patients with access to the interactive sections than for patients denied this access".

3b) CONSORT: Important changes to methods after trial commencement (such as eligibility criteria), with reasons

Not applicable

3b-i) Bug fixes, Downtimes, Content Changes

4a) CONSORT: Eligibility criteria for participants

" In each clinic and rehabilitation center, at least one healthcare provider was identified as a reference person who introduced the study to patients meeting predefined inclusion criteria. These were: (1) aged > 18 years, (2) having suffered from back pain for at least 3 months, (3) no concurrent involvement in other studies".

4a-i) Computer / Internet literacy

4a-ii) Open vs. closed, web-based vs. face-to-face assessments:

Web based assessment:
"Each participant logged in with a unique user ID so that no identifying information would be linked to their assessment, and the data were stored on secure servers. A password-protected document linking participant names to user IDs was maintained by the study coordinator, but this was not accessible to individuals involved in analyzing outcome data. Before granting access to the website, all participants were asked to complete an online questionnaire for baseline assessment. After 4 weeks, participants were asked to complete an online questionnaire for midterm assessment, and after 8 weeks this was repeated to get a final assessment.   "

4a-iii) Information giving during recruitment

4b) CONSORT: Settings and locations where the data were collected
"Participants were continually screened from February to June 2013 through their healthcare provider at selected clinics and rehabilitation centers in the Canton Ticino (Switzerland). " Please also refer to Fig.2
4b-i) Report if outcomes were (self-)assessed through online questionnaires
"Before granting access to the website, all participants were asked to complete an online questionnaire for baseline assessment. After 4 weeks, participants were asked to complete an online questionnaire for midterm assessment, and after 8 weeks this was repeated to get a final assessment. "
4b-ii) Report how institutional affiliations are displayed

5) CONSORT: Describe the interventions for each group with sufficient details to allow replication, including how and when they were actually administered
5-i) Mention names, credential, affiliations of the developers, sponsors, and owners

5-ii) Describe the history/development process

5-iii) Revisions and updating

5-iv) Quality assurance methods

5-v) Ensure replicability by publishing the source code, and/or providing screenshots/screen-capture video, and/or providing flowcharts of the algorithms used

5-vi) Digital preservation

5-vii) Access
"Each participant logged in with a unique user ID so that no identifying information would be linked to their assessment, and the data were stored on secure servers. A password-protected document linking participant names to user IDs was maintained by the study coordinator, but this was not accessible to individuals involved in analyzing outcome data."
5-viii) Mode of delivery, features/functionalities/components of the intervention and comparator, and the theoretical framework
"For this study, a modified version of the original website was created restricting access to contents on cBP only. A choice of static features including the library, the first aid section and a frequently asked questions (FAQ) section as well as interactive features including the virtual gym and the testimonials & commentaries sections were maintained from the ONESELF website [for a detailed description see 19,34,41-43]. In addition, two interactive features were newly developed and implemented: a weekly action plan and a quiz game. The weekly action plan required patients to select at the beginning of each week from a predefined list one or more physical activities of varying intensity to be completed during the week. Reminder SMS supported patients in complying with the plan. This feature was added based on insights of its effectiveness on chronic disease management from previous online and offline interventions [44-46]. The quiz game allowed patients to test the information learnt during their navigation of the website through multiple choice questions. For each correct answer patients earned virtual points. The sum of these points was used to rank patients in a classification list including all study participants. This form of interactivity through feedback is proposed in the context of gamification with the aim of using game thinking and game mechanics in non-game contexts to engage users in improving knowledge on cBP and patient empowerment [47]."
5-ix) Describe use parameters

5-x) Clarify the level of human involvement

5-xi) Report any prompts/reminders used
"The weekly action plan required patients to select at the beginning of each week from a predefined list one or more physical activities of varying intensity to be completed during the week. Reminder SMS supported patients in complying with the plan".
5-xii) Describe any co-interventions (incl. training/support)
Not applicable, we did not use any training/ support service
6a) CONSORT: Completely defined pre-specified primary and secondary outcome measures, including how and when they were assessed
"Outcome Measures
Empowerment was measured the with the Psychological Empowerment Scale [31] originally developed by Thomas and Velthouse model, already cited above. The scale was originally developed for use in the workplace setting and it was adapted to be used in healthcare setting [19, 34]. According to the authors, empowerment is a multidimensional concept composed of four cognitive dimensions (or task assessments): impact (or the degree to which behavior is seen as “making a difference”), competence (or the degree to which a person can perform task activities skillfully), meaningfulness (or the individual’s intrinsic caring about a given task), and choice (or whether a person’s behavior is perceived as self-determined) [49]. This conceptualization aims at psychological empowerment, that is the subjective impression that one has mastery over one’s health decisions. Incorporating the multidimensionality of the concept, the scale used in this study [48] consists of three items adopted to the context of cBP for each of the four sub-dimensions: meaning (e.g., “Dealing with my back pain is very important to me”), competence (e.g., “I am confident about my ability to do deal with back pain”), self-determination (e.g., “I have significant autonomy in determining how I deal with my back pain”) , and impact (e.g., “My control over the management of my back pain is large”). Participants responded on a Likert-scale ranging from 1 (strongly disagree) to 7 (strongly agree) with higher values suggesting higher levels of psychological empowerment. At all three assessment points, the four subscales presented good internal consistency with an alpha value ranging from 0.71 to 0.94.
Medication misuse was measured with the Prescription Medication Use and Perception of Risk Instrument [50]. The scale was scored as a continuous measure to provide greater sensitivity ranging from 0 (strongly disagree) to 6 (strongly agree) with higher scores indicating greater level of medication misuse.
Physical exercise in leisure time was measured with the respective sub-dimensions from the Short Questionnaire to Assess Health-Enhancing Physical Activity [51]. A sum score was calculated for the amount of time spent on physical exercise (hours) per week.
Pain burden was measured with six items from the Chronic Pain Grading Scale [52]. Three items measured pain intensity on an 11-point scale ranging from 0 (no pain) to 10 (pain as bad as it could be). Another three items measured pain disability on an 11-point scale ranging from 0 (no interference/ no change) to 10 (unable to carry on activities/ extreme change). Higher values imply worse health status. At all three assessment points, the two sub-scales presented good internal consistency with alpha values ranging from 0.74 to 0.92 except for the pain disability scale that obtained a lower internal consistency at the final assessment ($\alpha = 0.62$).
"
6a-i) Online questionnaires: describe if they were validated for online use and apply CHERRIES items to describe how the questionnaires were designed/deployed

6a-ii) Describe whether and how “use” (including intensity of use/dosage) was defined/measured/monitored

6a-iii) Describe whether, how, and when qualitative feedback from participants was obtained

6b) CONSORT: Any changes to trial outcomes after the trial commenced, with reasons
Not applicable, no changes occured after the trial commenced
7a) CONSORT: How sample size was determined
7a-i) Describe whether and how expected attrition was taken into account when calculating the sample size

7b) CONSORT: When applicable, explanation of any interim analyses and stopping guidelines
Not applicable, interim analysis was not planned
8a) CONSORT: Method used to generate the random allocation sequence
"After confirming eligibility and obtaining informed consent from the patient (via email), the study coordinator randomly allocated participant to the two-armed parallel groups using a freely available computerized random number generator program. A permuted block randomization design method was used during the 3-month enrollment period to ensure roughly equal numbers of patients were allocated to each group. There was no face-to-face contact between the patients and research team at any point in the study, which allowed participants to live anywhere in Canton Ticino (Southern Switzerland). Of the 51 participants; 27 were allocated to the intervention group and 24 to the control group. "
8b) CONSORT: Type of randomisation; details of any restriction (such as blocking and block size)
"A permuted block randomization design method was used "
9) CONSORT: Mechanism used to implement the random allocation sequence (such as sequentially numbered containers), describing any steps taken to conceal the sequence until interventions were assigned
"the study coordinator randomly allocated participant to the two-armed parallel groups using a freely available computerized random number generator program"
10) CONSORT: Who generated the random allocation sequence, who enrolled participants, and who assigned participants to interventions
"the study coordinator"
11a) CONSORT: Blinding - If done, who was blinded after assignment to interventions (for example, participants, care providers, those assessing outcomes) and how
11a-i) Specify who was blinded, and who wasn’t
"Patients were blinded to the arm to which they were randomized."
11a-ii) Discuss e.g., whether participants knew which intervention was the “intervention of interest” and which one was the “comparator”

11b) CONSORT: If relevant, description of the similarity of interventions
Not applicable
12a) CONSORT: Statistical methods used to compare groups for primary and secondary outcomes
"Testing H1, that is the improvement in patient empowerment and physical exercise as well as the decrease in medication misuse and pain burden, the development of self-report measures over the three assessment points is looked at. Changes over time are analyzed with paired samples t-tests. Testing H2, that is the stronger improvement in patient empowerment and physical exercise as well as the stronger decrease in medication misuse and pain burden in the intervention group over the control group makes use of the randomized controlled study design and potentially yields strong evidence for the incremental effect of interactive features over merely static informational features. Differences between the two versions of the intervention (static vs. interactive) are analyzed with independent samples t-tests and with Chi-square tests for categorical variables. Differences in change over time are not determined on the aggregate but on the individual level and then averaged. This allows the use of t-tests for significance testing".
12a-i) Imputation techniques to deal with attrition / missing values
" Differences between the two versions of the intervention (static vs. interactive) are analyzed with independent samples t-tests and with Chi-square tests for categorical variables. Differences in change over time are not determined on the aggregate but on the individual level and then averaged. This allows the use of t-tests for significance testing"
12b) CONSORT: Methods for additional analyses, such as subgroup analyses and adjusted analyses
"Differences between the two versions of the intervention (static vs. interactive) are analyzed with independent samples t-tests and with Chi-square tests for categorical variables. Differences in change over time are not determined on the aggregate but on the individual level and then averaged. This allows the use of t-tests for significance testing"

RESULTS

13a) CONSORT: For each group, the numbers of participants who were randomly assigned, received intended treatment, and were analysed for the primary outcome
"Table 1 compares participants’ socio-demographics divided by intervention and control group. There were no significant differences for any patient characteristics, although there was a trend towards higher education among participants of the intervention group". Please also refer to Table 1
13b) CONSORT: For each group, losses and exclusions after randomisation, together with reasons
Please refer to Fig.2
13b-i) Attrition diagram

14a) CONSORT: Dates defining the periods of recruitment and follow-up
Please refer to Fig.2
14a-i) Indicate if critical “secular events” fell into the study period

14b) CONSORT: Why the trial ended or was stopped (early)
Not applicable, the study was not stopped early
15) CONSORT: A table showing baseline demographic and clinical characteristics for each group
Please refer to Table 1
15-i) Report demographics associated with digital divide issues
Please refer to Table 1
16a) CONSORT: For each group, number of participants (denominator) included in each analysis and whether the analysis was by original assigned groups
16-i) Report multiple “denominators” and provide definitions
Please refer to Table 2

16-ii) Primary analysis should be intent-to-treat

17a) CONSORT: For each primary and secondary outcome, results for each group, and the estimated effect size and its precision (such as 95% confidence interval)
Please refer to Table 2
17a-i) Presentation of process outcomes such as metrics of use and intensity of use

17b) CONSORT: For binary outcomes, presentation of both absolute and relative effect sizes is recommended
Please refer Table 2
18) CONSORT: Results of any other analyses performed, including subgroup analyses and adjusted analyses, distinguishing pre-specified from exploratory
Please refer to Table 2
18-i) Subgroup analysis of comparing only users

19) CONSORT: All important harms or unintended effects in each group
Not applicable, no adverse events occurred
19-i) Include privacy breaches, technical problems

19-ii) Include qualitative feedback from participants or observations from staff/researchers

DISCUSSION

20) CONSORT: Trial limitations, addressing sources of potential bias, imprecision, multiplicity of analyses
20-i) Typical limitations in ehealth trials
"This randomized controlled study is not without any limitations, which are mainly of methodological nature. Firstly, the study suffers from a small group size, despite the significant differences found between the two conditions. A bigger sample size might have strengthened marginally significant results and helped to detect significant differences within the intervention group for physical exercise. Secondly, the study lacks a pure control group. In fact, patients provided with the static version of the website were used as a control group, but no group of cBP patients was included with no access to the Internet-based intervention at all. However, the main objective of this study was to test the effectiveness of interactive sections compared to static elements only and not to test the effectiveness of the intervention as a whole. Third, a two--months intervention might be too short a period to discover meaningful effects and conclude on the effectiveness of Internet-based interventions on maintaining high levels of empowerment and beneficial self-management behaviors. Eventually, the present study lacks specificity inasmuch it did not take into account the quality of any of the sections that might have caused the differences between the intervention group with interactive sections and the control group with static elements only. "
21) CONSORT: Generalisability (external validity, applicability) of the trial findings
21-i) Generalizability to other populations

21-ii) Discuss if there were elements in the RCT that would be different in a routine application setting

22) CONSORT: Interpretation consistent with results, balancing benefits and harms, and considering other relevant evidence
22-i) Restate study questions and summarize the answers suggested by the data, starting with primary outcomes and process outcomes (use)

"Considering one of its main objects, that is understanding the impact of Internet-based interventions like ONESELF on patient empowerment, the study found a differential effect for the two experimental conditions. Among patients without access to the interactive sections, empowerment remained constant after eight weeks while it significantly increased and remained consistently higher among patients who had access to the interactive sections. This suggests that the interactive sections of healthcare websites might indeed play a role in empowering patients with chronic conditions and gives useful insights compared to studies with contradictory results that did not pay attention to the presence or absence of interactive website features. Further evidence for the empowering effect of interactive features could be gained by looking at the actual use of these as we would expect heavy users of interactive features to demonstrate a larger increase in empowerment than light users of these features. Future studies are needed to test this hypothesis. The differential effects of the website versions on patient empowerment refer to an overall score across all four dimensions of psychological empowerment. But they hold for each of the four dimensions, too. This suggests, beyond the analyses of the psychometric qualities of this scale, that the four dimensions indeed belong together and contribute to the overall concept of empowerment. Considering the four dimensions separately, patients in the intervention group significantly improved their perceived self-determination, meaningfulness and competence.

With regards to the differential effect of the website versions on self-management behaviors related to cBP, the results of this study show a considerable decline of physical exercise at both the midterm and the final assessment, irrespective of the experimental condition. One explanation could be that the use of websites like ONESELF, independent of the presence of interactive features, prevents people from doing what is good for them, that is in this case exercising to relieve pain. This, however, would run against the explicit objectives and contents of the Internet-based intervention, which put great emphasis on the necessity of exercising (the website used weekly action plans with reminder SMS aimed at motivating cBP patients to engage in regular physical exercise); and it would also run against the findings of other studies [53-55]. Another more probable explanation could be that the results are due to measurement effects. Indeed, the study might have been affected by seasonal effects related to the period of enrollment since almost half of the participants (N = 22) reported on their physical exercise in July and August, which are both popular holiday months in Switzerland where many people interrupt their habitual activities including physical exercise.

Results show that, overall, medication misuse did not change much as a result of the Internet-based intervention. That, however, hides very different developments in the two experimental groups: While misuse went up in the control group, it went down in the group with access to the interactive features, even though the difference between midterm and final assessment is not significant. Increased medication misuse as a consequence of a healthcare website is difficult to interpret but cannot be completely ruled out. No matter where the increased misuse in the control group may originate from, interactivity appears to have the potential to work against that, at least in keeping control over the use of such medications and adhering to medical regimes.

Eventually, participants experienced less pain as the exposure to the Internet-based intervention proceeded. If the intervention contributed to this decline, it was not due to its interactive features as the decrease in pain burden was observed in both groups. Strangely enough we observed, over the course of the experiment, a reduction in physical exercise but a clear improvement of the pain condition. The most straightforward interpretation of this aggregate result would be that, contrary to most assumptions, the relationship between exercise and pain is different than expected. But to posit a positive relationship—more exercise, more pain—would certainly be premature, if for the fact alone that the increased misuse of medication among the control group would be difficult to explain. Both developments could be, however, again explained by seasonal effects. Measurement in summer might be responsible for both low levels of physical exercise due to a break of habitual behaviors for holidays, and lower levels of pain than in other times of the year with cold and rainy weather. Moreover, a lower level of back pain might be ascribed to a diminishment of work and work-related stress that can contribute to a decreased level of pain [56- 57].

Back pain patients with access to the static elements of ONESELF providing information only gave up on their exercise, felt less pain, and reported more medication misuse. Participants of the intervention group with access to the interactive elements on top of the informative ones also gave up on their exercise, also felt less pain, but reported less medication misuse. These patients felt more empowered through the Internet-based intervention as, compared to patients of the control group, they reported to have better mastery over their cBP at the end of the intervention. We can therefore conclude that the interactive features of the ONESELF website indeed contributed to improving patient empowerment while purely static elements with information only did not. Hence, this study complements the emerging literature supporting the utility of Internet-based interventions aimed at patient empowerment. The empowered patient emerges as a person who does not passively receive information, but takes increased responsibility for and a more active role in decision making regarding his or her health [27-30]. This study highlights how empowerment is strengthened by interactivity, and this result enhances the existing literature in the field about the conjunction of these two constructs [35-37]".

22-ii) Highlight unanswered new questions, suggest future research

"Further insights on which specific elements cause change are essential to better inform the design of future Internet-based interventions aimed at improving chronically ill patients' empowerment, self-management behaviors and, ultimately, their health status"

Other information

23) CONSORT: Registration number and name of trial registry

Trial Registration: www.ClinicalTrial.gov NCT02114788; Information available at:
http://www.clinicaltrials.gov/ct2/show/NCT02114788?term=NCT02114788&rank=1

24) CONSORT: Where the full trial protocol can be accessed, if available

Protocol is not available. Information are available at:
http://www.clinicaltrials.gov/ct2/show/NCT02114788?term=NCT02114788&rank=1

25) CONSORT: Sources of funding and other support (such as supply of drugs), role of funders

This project was supported by a grant from the Swiss National Science Foundation (FNSNF), Project Funding Number: FNS100013_130030/1.

X26-i) Comment on ethics committee approval

x26-ii) Outline informed consent procedures

X26-iii) Safety and security procedures

X27-i) State the relation of the study team towards the system being evaluated

The authors stated that they had no interests which might be perceived as posing a conflict or bias
